# “You Are on the Right Track With the App:” Qualitative Analysis of Mobile Phone Use and User Feedback Regarding Mobile Phone Sexual Risk Assessments for HIV Prevention Research

**DOI:** 10.3389/fdgth.2021.576514

**Published:** 2021-03-22

**Authors:** Janan J. Dietrich, Gabriella L. Benadé, Mamakiri Mulaudzi, Ashraf Kagee, Stefanie Hornschuh, Lerato M. Makhale, Maria P. Lemos, Erica Lazarus, Michele P. Andrasik, Keith J. Horvath

**Affiliations:** ^1^Perinatal HIV Research Unit, School of Clinical Medicine, Faculty of Health Sciences, University of the Witwatersrand, Johannesburg, South Africa; ^2^Health Systems Research Unit, South African Medical Research Council, Bellville, South Africa; ^3^Department of Psychology, Faculty of Arts and Social Sciences, Stellenbosch University, Stellenbosch, South Africa; ^4^Vaccine and Infectious Disease Division, Fred Hutchinson Cancer Research Center, Seattle, WA, United States; ^5^Department of Psychology, San Diego State University, San Diego, CA, United States

**Keywords:** digital health, mobile health, daily diaries, e-health, sexual risk assessment, momentary ecological assessments, HIV, Africa

## Abstract

**Background:** Accurate self-report of sexual behavior assists in identifying potential HIV exposure in HIV prevention trials. Brief mobile phone assessments, completed daily or after sexual activity, can improve the validity and reliability of self-reported sexual behavior and allow for remote survey completion outside of the clinic setting. We conducted a qualitative study to better understand participants mobile phone use and to explore their perspectives on how to improve an existing mobile application-based sexual risk assessment.

**Methods:** Sexually active, HIV seronegative men (*n* = 14) and women (*n* = 15) aged 18–39 years were recruited through an HIV counseling and testing clinic and community outreach in Soweto, South Africa. We conducted qualitative research through four age-stratified focus group discussions (FGDs) and analyzed a brief socio-demographics and mobile phone access questionnaire. All participants completed a sexual risk assessment before the FGD. Using a framework analytic approach, data were coded with Nvivo software.

**Results:** All participants had access to mobile phones and internet, and 27 (93.1%) were able to download applications on their personal phones. Participants preferred mobile risk assessments to be offered in a choice of South African languages, using formal language (as opposed to emojis), with straight-forward wording and limited to five to 10 questions. Most participants found it acceptable to complete the assessment once a week, on a weekday, while a few were willing to complete it after each sexual encounter. It was suggested that a message reminder to complete the assessment should be sent at least daily until it is completed. The majority agreed that a password-protected application with a discreet logo was ideal for privacy, ease of use and flexibility for completion in any setting. A concern with this format, however, was the potential data use requirement. Participants expressed privacy concerns with using SMS, WhatsApp and other social media for risk assessments. Most agreed on an airtime incentive between ZAR5-10 (USD 0.29–0.58) per survey. Participants encouraged researchers to provide feedback to them about their sexual risk.

**Conclusions:** Completion of mobile phone sexual risk assessments can be optimized with minimal incentives by ensuring that questionnaires are simple, brief, infrequent and have trusted privacy measures.

## Introduction

South Africa (SA) carries 16% of the global burden of Human Immunodeficiency Virus (HIV), with an HIV prevalence of 12% (1). In HIV prevention trials—including vaccines, oral or injectable Pre-Exposure Prophylaxis (PrEP), and microbicides—identifying HIV exposure through sexual activity is an ongoing challenge (2–5). In the absence of a biological marker to identify actual HIV exposure, retrospective self-reported risky sexual behavior is usually used as a proxy of potential exposure to infection. Self-report data collection methods include face-to-face interviewing, daily diaries, computer-assisted self-interviews (CASI) and audio computer-assisted self-interviews (ACASI). However, none of these have been identified as a gold standard for data collection of sexual activity (6–8).

Face-to-face interviewing conducted at the clinic is the most commonly used method of behavioral risk assessment in SA, but it is prone to bias (9). Measurement error related to self-report is common, due to social desirability response bias and recall error (10–13) given long recall periods and the challenges associated with capturing complex patterns of sexual activity (14–17). Interviewer-administered in-clinic assessments are often time-consuming, resulting in response fatigue by interviewers and participants, which may prompt some researchers to utilize ACASI. However, ACASI may be problematic as it is initially expensive to purchase the equipment and is only cost-effective if it is used for studies with large sample sizes or across many different studies (18). Participants also need to be able to use the software. Many of these challenges may be mitigated with a brief, mobile phone assessment, completed daily or prompted through sexual activity. This method may improve the validity and reliability of self-reported sexual behavior as it allows for more anonymous, remote (away from the clinic) real-time reporting.

Mobile phone use is ubiquitous (19, 20) but it is unknown whether smartphones with internet and application (app) capacity are as ubiquitous in settings like Soweto, South Africa. At least two thirds of the general SA population have access to a mobile phone (21, 22). Mobile phone interventions have been shown to be feasible tools in HIV prevention trials in SA, especially among at-risk populations (23, 24). In a study conducted by Dietrich et al. (23), 50 adult women in Soweto, South Africa completed daily mobile phone questionnaires over 12 weeks and showed an overall response rate of 82%. The study also showed that self-report of vaginal sexual intercourse was significantly higher through the mobile phone surveys than the in-clinic questionnaires. Overall, the study showed that collecting sexual behavioral data via mobile phone is feasible. Another study conducted in Kenya by Curran et al. (24) used a short message service (SMS) method to set up surveys to collect data on sexual behavior and pre-exposure prophylaxis (PrEP) adherence. Ninety-six HIV uninfected adults took PrEP daily and responded to SMS surveys for 60 days. Overall, 94% of surveys were successfully answered, which showed a high response rate and that using a mobile phone based survey method to collect sensitive sexual health and behavior information is feasible and acceptable. There are certainly few published data within the area of implementing remote data collection within South African HIV prevention research. Even fewer studies engage potential users in the design and development of health applications. We address this gap of user engagement through obtaining user feedback which are to be used to optimize the app for future implementation. Therefore, we conducted a qualitative study to determine mobile phone use among adults aged 18–39 years seeking HIV counseling and testing at a community outreach clinic, as well as to explore their perspectives on how to improve upon an existing mobile phone sexual risk assessment. The current study extends the work of HVTN 915 by obtaining user recommendations on the mobile phone assessment used in the HVTN 915 prospective cohort study of 50 adult women 18–25 years in Soweto, South Africa who were followed up for 12 weeks (23, 25). Participants of HVTN 915 were provided with internet-enabled smartphones to complete surveys consisting of five daily sexual behavior questions via SurveyCTO, an independent mobile phone app. SurveyCTO was pre-loaded on the mobile device prior to providing the phone to the participant. Participants also completed eight interviewer-administered in-clinic behavioral risk questionnaires during the study. In addition, vaginal swabs were self-collected daily and used to assess occurrence of unprotected sex and possible exposure to HIV through detection of Y chromosome and HIV, respectively. Results from a comparison of mobile phone assessments to in-clinic questionnaires are reported in more detail elsewhere (23, 25). Briefly, sex acts reported via the mobile phone app were more accurate than *via* the in-clinic questionnaire (25), suggesting that daily mobile phone surveys reduced the social desirability and recall biases on in-clinic administered questionnaires. In addition, the results showed high adherence to the daily mobile phone survey completion with an 82% response rate of 4219 delivered surveys (23). A limitation of the HVTN 915 design was that the sample did not include men and people older than 25 years and was therefore not representative of current HIV prevention trial target populations (26, 27). For the present study we obtained user recommendations among 19–39-year-old men and women to optimize the HVTN 915 mobile phone sexual risk assessment for use in HIV vaccine prevention trials. We facilitated discussions with the participants in which we explored preferences for format, language, frequency of survey completion, frequency and timing of reminders and incentives for the completion of the mobile phone survey. Additionally, we explored facilitators and barriers to mobile phone surveys.

## Methods

We conducted a qualitative study, between June 2018 and November 2018, consisting of a brief socio-demographics and mobile phone use questionnaire (22, 28) and a qualitative component consisting of four focus group discussions (FGDs) using a semi-structured interview guide (29–31). Qualitative research is particularly important for engaging participants for obtaining user feedback.

### Setting

The study was conducted at the Perinatal HIV Research Unit (PHRU) located at the Chris Hani Baragwanath Academic Hospital in Soweto, Johannesburg. The PHRU has conducted research in the Soweto community for more than 20 years. The PHRU vaccines research unit was the first site in South Africa to conduct an HIV vaccine trial with multiple completed and ongoing large-scale HIV vaccine trials (32).

### Participant Sampling and Recruitment

We used a convenience sampling strategy to recruit men and women who were 19–39 years old, sexually active, HIV-uninfected, and residing in Soweto. Participants were recruited through ZAZI, the PHRU HIV counseling and testing (HCT) clinic. Prospective participants were referred from ZAZI to the study team if they had a confirmed HIV negative rapid test within 3 months at the time of the study and if they reported being sexually active (defined as two or more sex acts per week). Our study team were able to approach participants in-person, if they were still at the clinic or telephonically to provide a brief overview of the study and to establish initial interest. Those that expressed interest were invited to return to the clinic for the scheduled FGD. The FGD was conducted at a private room at the PHRU. Upon arrival for participation in the FGD, participants were screened using their identity documentation to confirm that they met the age criteria of the study. Participants who met the eligibility criteria then provided written informed consent were then screened around their sexual activity.

### Procedures

Experienced and multi-lingual qualitative research interviewers conducted all the procedures related to the four (two with men and two with women) age-stratified (19–30 and 31–39 years) FGDs. The FGDs were conducted in private rooms at the PHRU. The FGDs consisted of an average of seven (range 6–8) participants per FGD and lasted on average 94.25 minutes.

**Figure 1:**
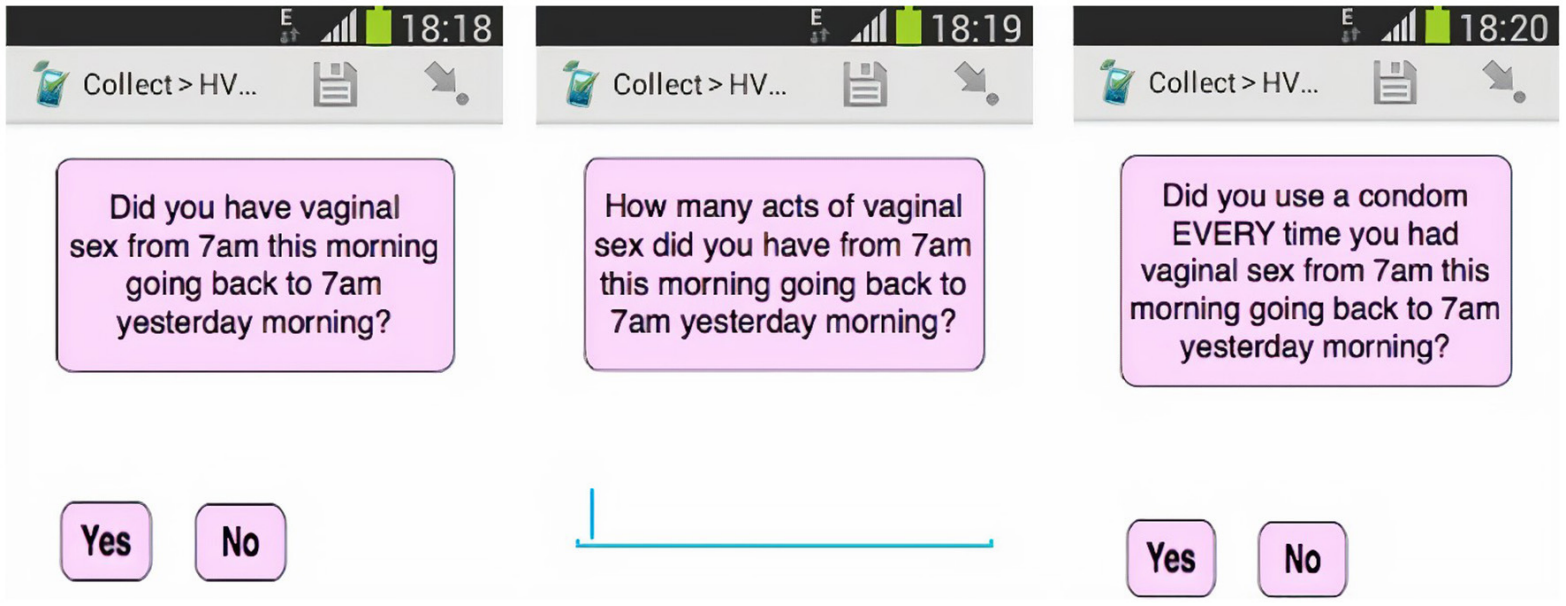
Mobile phone risk assessment given to participants prior to the FGD. Reprinted from Dietrich et al. (23) reprinted with permission.

The procedures for the study participation took place in the following chronological sequence consisting of four main steps. Prior to the FGD discussion (1) participants provided written informed consent, and (2) were shown the smartphone that was already pre-loaded with the SurveyCTO sexual risk assessment survey questions used in HVTN 915 (Figure 1). At this stage, participants could navigate through the sexual risk assessment questions on the smartphone and scroll through the questions and response options to see how the SurveyCTO application worked and how the questions were presented. This step would have assisted the participants in answering some of the questions that would be addressed when they participated in the FGD. Participants were not required to complete the sexual risk assessment survey as part of participation in the FGD (3). Participants then completed a brief questionnaire on socio-demographics (Table 1) and mobile phone use (Table 2) (4). Finally, the qualitative researchers conducted the FGDs using a semi-structured interview guide.

**Table 1:** Socio-demographic questionnaire.

**Questions**	**Response options**
Age	18-39
Gender	Male Female
Main language spoken in your home?	Afrikaans IsiZulu SiSwati English Northern Sotho Tshivenda IsiNdebele Sesotho Xitsonga IsiXhosa Setswana other
What is the main material that the walls of your house are built of?	Brick house owned by family Brick house that family is renting Flat owned by family Flat that family is renting RDP house Hostel-brick Shack-informal settlement Shack-backyard Other
What is the highest level of formal education you have completed?	No formal education, Incomplete primary school (up to grade 7) Complete primary school (completed grade 7) Incomplete high school (up to grade 12) Complete high school (completed grade 12) Incomplete post-high school training (trade or technical training, college, or university) Complete post-high school training (trade or technical training, college, or university) Other

**Table 2:** Mobile phone use questionnaire.

**Question**	**Response options**
Do you own a personal mobile phone?	Yes No
If yes, do you share your personal phone with someone?	Yes No
If no, do you use someone else's mobile	Yes No
If you share your phone, who do you share it with?	Parents Partner Sibling(s) Friend(s) Grandparents Cousin Other
What type of phone do you have/share?	Samsung BlackBerry Nokia Sony LG HCT Huawei Motorola Other
Please specify the make	Open ended response
Can you access internet on the phone?	Yes No
Can you download applications with the phone?	Yes No
How much time in a day do you spend actively using a mobile phone? This includes using it for listening to music/radio, SMS, making phone calls, playing games and accessing the internet.	0–1 h 2–4 h 5–7 h More than 8 h Don't know
How do you get airtime?	Prepaid Contract I don't get airtime Don't know
Who pays for the airtime?	Parent Friend Partner My own money Sibling Other
How do you get data bundles?	Prepaid Contract I don't get data Don't know
Who pays for the data?	Parent Friend Partner My own money Sibling Other
In the past year, was your phone service ever disconnected because you could not pay the bill, afford airtime or because your phone was lost or stolen?	Yes No
In the last 6 months, have you had access to the internet?	Yes No
How do you mainly access the internet? (please choose only one)	Mobile phone Laptop Personal Computer Tablet Other
Do you have an active Facebook profile?	Yes No

### Measures

The socio-demographic and mobile phone use questionnaire (22, 28) was designed by the study team and assessed questions that are detailed in Tables 1 and 2, respectively. Interviewers used a semi-structured interview guide (29–31) that was designed by the study team to facilitate discussion on the optimization of the mobile phone survey. The interview guide prompted questions about preferences for the mobile phone survey formatting, content of questions, language/s to be used, frequency and timing of reminder messages and incentives for participants to complete the mobile phone survey. Furthermore, the interview guide included questions about perceived barriers and facilitators that participants would encounter in responding to a mobile phone sexual risk assessment survey in the long term.

### Ethical Considerations

Study procedures were approved by the University of the Witwatersrand Human Research Ethics Committee. Participants provided written informed consent for participation and received ZAR100 (~USD 6 at the time of writing) reimbursement. Pseudonyms were used during the FGDs to ensure confidentiality and no personal identifying information were used in the audio-recordings.

### Data Preparation and Analysis

#### Quantitative Data Analyses

The questionnaire data were entered into an excel spreadsheet and analyzed using Microsoft excel. The participants age was described using median and interquartile ranges and the descriptive data was summarized using counts and percentages.

#### Qualitative Data Analyses

FGDs were audio-recorded and transcribed verbatim. Initial coding was conducted by a primary analyst, a trained qualitative researcher, and presented to the senior author for review and feedback. Thereafter, another trained analyst and two experienced analysts conducted a thematic analysis using both a deductive and inductive approach (33). First, a coding framework using a table in Microsoft word was created according to questions in the FGD guide. The first FGD transcript was coded using a line-by-line analysis to assign text to codes allowing unexpected codes to emerge. The first author reviewed the code-book and addressed discrepancies. Emerging codes were added to the coding framework to create a codebook. The codes and transcripts were then imported into Nvivo (QSR International Pty Ltd. Version 12. 2018). The next three FGDs were coded in Nvivo using axial coding. Once coding was complete, codes were examined to identify patterns, common concepts and emerging ideas. Identified patterns and concepts were grouped according to categories to develop themes. To reduce subjectivity in the coding and analysis process, final interpretation was conducted. Access to and use of mobile phones were examined to inform the development and optimization of a mobile phone sexual risk assessment that could be integrated into the participant's daily mobile phone practices. Next, key recommendations for optimizing a mobile sexual risk assessment were explored, followed by facilitators and barriers to using this assessment.

## Results

### Participant Characteristics

Twenty-nine participants were enrolled in the FGDs, including 15 women (51%). The median age of participants was 30 [interquartile range (IQR) 24–34] and 12 (41%) spoke isiZulu. Twenty-four (83%) participants lived in brick houses and five (17%) lived in shacks (informal small dwelling, makeshift and not built according to approved construction plans, usually hand constructed using any freely available materials) (34). Twenty-two (75.9%) completed high school (grade 12) of which nine had advanced to tertiary level studies (Table 3).

**Table 3:** Quantitative results from the socio-demographics and mobile phone use questionnaire.

**Variable**	**Female (*n* = 15)**	**Male (*n* = 14)**	**Total (*n* = 29)**
**Median age (IQR)**	30 (IQR 23–33)	29.5 (IQR 26.5–36.5)	30 (IQR 24.25–33.75)
**Home language**, ***n*** **(%)**			
IsiZulu	6 (40)	6 (42.9)	12 (41.4)
IsiXhosa	1 (6.7)	4 (28.6)	5 (17.2)
Sesotho	4 (26.7)	0 (0.0)	4 (13.8)
Other (Setswana, IsiNdebele, Tshivenda, Xitsonga, Northern Sotho, Afrikaans)	4 (26.7)	4 (28.6)	8 (27.6)
**Dwelling**, ***n*** **(%)**			
Brick house	12 (80.0)	12 (85.7)	24 (82.8)
Shack	3 (20.0)	2 (14.3)	5 (17.2)
**Highest level of formal education**, ***n*** **(%)**			
High school (grade 12) not completed or attended	1 (6.7)	6 (42.9)	7 (24.1)
High school (grade 12) completed	12 (80.0)	1 (7.1)	13 (44.8)
Studying or completed post-high school training (Trade or technical training, college, or university)	2 (13.3)	7 (50.0)	9 (31.0)
**Do you own a personal mobile phone? *n* (%)**			
Yes	14 (93.3)	13 (92.9)	27 (93.1)
No	1 (6.7)	1 (7.1)	2 (6.9)
**What type of phone do you have/ share? *n* (%)**			
Huawei	2 (13.3)	1 (7.1)	3 (10.3)
Samsung	4 (26.7)	4 (28.6)	8 (27.6)
Nokia	0 (0.0)	2 (14.3)	2 (6.9)
Mobicell	3 (20.0)	3 (21.4)	6 (20.7)
Vodafone	3 (20.0)	0 (0.0)	3 (10.3)
Other (Alcatel, Mobiwire, STK p5, Hisense, Sony)	3 (20.0)	4 (28.6)	7 (24.1)
**Access to internet on the phone**, ***n*** **(%)**	15 (100.0)	14 (100.0)	29 (100.0)
**Ability to download applications on the phone (*n* = 27), *n* (%)**	14 (93.3)	13 (92.9)	27 (93.1)
**How much time in a day do you spend actively using a mobile phone? *n* (%)**			
<4 h	3 (20.0)	7 (50.0)	10 (34.5)
>4 h	11 (73.3)	5 (35.7)	16 (55.2)
**How do you get airtime? *n* (%)**			
Prepaid	14 (93.3)	13 (92.9)	27 (93.1)
Contract	0 (0.0)	1 (7.1)	1 (3.4)
I don't get airtime	1 (6.7)	0 (0.0)	1 (3.4)
**Who pays for the airtime? *n* (%)**			
My own money	10 (66.7)	12 (85.7)	22 (75.9)
Friend / parent / partner	5 (33.3)	2 (14.3)	7 (24.1)
**How do you get data bundles? *n* (%)**			
Prepaid	15 (100.0)	14 (100.0)	29 (100.0)
**Who pays for the data? *n* (%)**			
My own money	10 (66.7)	12 (85.7)	22 (75.9)
Parent / Partner	5 (33.3)	2 (14.3)	7 (24.1)
**In the past year, was your phone service ever disconnected because you could not pay the bill, afford airtime or because your phone was lost or stolen? (*n* = 28), *n* (%)**	(*n* = 15)	(*n* = 13)	
Yes	5 (33.3)	4 (30.8)	9 (32.1)
No	10 (66.7)	9 (69.2)	19 (67.9)
**Access to the internet in the last 6 months, *n* (%)**	11 (73.3)	12 (85.7)	23 (79.3)
**How do you mainly access the internet? (*n* = 28), *n* (%)**	(*n* = 14)	(*n* = 14)	
Mobile phone	12 (85.7)	13 (92.9)	25 (89.3)
Laptop / PC / tablet	2 (14.3)	1 (7.1)	3 (10.7)
**Active Facebook profiles (*n* = 27), *n* (%)**	(*n* = 15)	(*n* = 12)	
Yes	7 (46.7)	9 (75.0)	16 (59.3)
No	8 (53.3)	3 (25.0)	11 (40.7)

### Access to and Use of Mobile Phones

All participants had access to mobile phones; 27 (93.1%) owned a mobile phone and two (6.9%) had access via shared phones. Six participants (23%) who owned phones shared them with family or friends. All participants had access to the internet on their phones and 27 (93.1%) were able to download mobile phone apps. On average, 16 (55.2%) spent more than 4 h actively on their phone (11 women and five men) (see Table 3).

The qualitative data revealed that participants used mobile phones for communication, social media, internet search engines and other applications for games and educational tools. Communication channels included phone calls, WhatsApp, and SMS. Some of the mentioned uses of internet search engines were to inform self-diagnosis and treatment of medical conditions including HIV, finding information on work related issues, getting advice on communication in a relationship, helping with their children's homework, learning about the latest news and seeking employment. Participants reported using Facebook, Twitter, and Instagram.

“All the answers I want I find them on Google, so I can't live without internet.” (female, aged 31–39)

“We always get phones that allow Facebook and WhatsApp.” (female, aged 19–30)

The quantitative data showed that 27 (93.1%) participants bought airtime for voice calls using common South African prepaid services and all participants received data bundles with prepaid packages. Participants reported data to be expensive with unreliable internet connection. Free Wi-Fi was one of the ways in which the participants mitigated the need for data. Participants accessed Wi-Fi from the library, restaurants or work. The disadvantages of free Wi-Fi access were increased risk of phone theft and difficulty connecting, as shown in the following quotes:

“Another thing that is a disadvantage [is] when the weather is otherwise [poor/adverse weather], you cannot connect and when there's a lot of people because some of them come with laptops. So if they do that that means you will struggle [to connect].” (female, aged 31–39)

“So when you (are) busy, busy with survey wherever you are, obvious someone can come and grab your phone while you are busy connecting the WIFI” (male, aged 19–30)

Although the quantitative data showed that all participants had access to the internet on their mobile phones, only 23 (89.3%) had access to the internet in the last 6 months and nine (32.1%) had their phone disconnected in the past year due to being unable to pay the phone bill, to afford airtime or because the phone was lost or stolen. This was supported by qualitative data from one of the FGD.

“I was having a Huawei and they stole it” (female, aged 31–39)

“I was using Samsung. Yes, it got stolen” (female, aged 31–39)

Through the qualitative data, participants mentioned that they used either Bluetooth or an application called “Share it” to share content in order to avoid the need for internet. “Share it” is a free mobile application that, once downloaded, is used to share large files *via* Bluetooth with another mobile phone user who also has the application.

Themes related to key recommendations for optimizing a mobile phone sexual risk assessment survey were explored, followed by identified facilitators and barriers to using this mobile phone survey in the long term, as would be the case in a HIV prevention clinical trial.

### User Recommendations for Optimizing the Existing Mobile Phone Survey

#### Design and Format

The majority of participants agreed that an application would be the best platform on which to deliver the mobile phone survey by offering privacy, ease of use, ease of access and separation from promotional market surveys that are sent to participant's phones.

“You are on the right track with the app [application]… you download it, you [researchers] send airtime. That's how surveys are done.” (male, aged 19–30)

“An app [application] is simple so let's just stick with the app [application] and not SMS” (male, aged 31–39)

“The good thing about the app is that you are not talking you are typing. It's a yes or no answer. You can do it while your boo [partner] is driving, you can do it as you are sitting as a family. You can do it right where you are.” (female, aged 19–30)

The data required for an application was a downside to this suggested platform,

“You should create something that doesn't want internet ‘cause nowadays its very expensive data so I think if you do the app that doesn't want internet it's going to be more reachable.” (male, aged 19–30)

“You are going to use data and I don't have access to data all the time. So if I'm going to have to answer every day then I'm going to need airtime. It's going to be a bit tricky” (male, aged 31–39)

Participants mentioned that if an application were used, the design of the app icon would need to be inconspicuous so as to not give away the purpose of the assessment.

“It must not be obvious ‘cause when you put something there and it relates to a sex app [application] and my mom would be like a ‘sex app [application]!” (female, aged 31–39)

A few participants suggested using WhatsApp or SMS to deliver the mobile phone survey:

“Maybe a survey like that through WhatsApp ‘cause WhatsApp is like our easiest way to communicate.” (female, aged 19–30)

One participant (male, aged 19–30) suggested that both an application and SMS or WhatsApp formats should be available depending on whether a participant has a smartphone or basic phone.

Only a few participants suggested the use of social media to deliver the survey using a Facebook or Twitter page because these platforms were used daily and therefore it would be possible to reach more people. Concerns about using a social media platform were lack of privacy and the sentiment that social time and survey time should be separate.

“I basically think it's a bomber to have a survey on social media because who would want to participate in that? An app [application] is basically all there could be. Because of why would you want to have a survey on social networks? … And basically, mix and match it doesn't click. It's a social time and it's a survey time. It doesn't mix it doesn't come together.” (male, aged 19–30)

“Doing surveys on social media? … [It] will not be published on my timeline, right? That would be better, on my timeline no!” (male, aged 19–30)

One suggestion was to use unstructured supplementary service data (USSD), a tool commonly used to buy airtime on mobile phones, and another was to adapt it into a game that would involve going up a level every time the participants answered a question.

#### Language and Wording

The majority of participants agreed that the mobile phone survey should be offered in a choice of different South African languages for accessibility. Most participants preferred the use of formal language.

“To get the message across, formal for me is simple and clear. I mean you don't go to school and get a text book with emojis.^1^” (male, aged 31–39)

“It's better formal than informal, you see, because informal for others it's not easy to understand: emojis, LOL, wow. Ha ah some people won't understand.” (male, aged 19–30)

However, three participants suggested that there could be an option between formal and informal language, because participants of different ages might have different preferences.

“Just include age at the beginning of the survey to say we cater for millennials and those born [earlier]. That way I think if, if you are used to text it is much more interesting. The questions are asked in a way that you best understand them.” (male, aged 19–30)

Two participants opted for informal language and another mentioned that it would depend on the person taking the survey. Some participants reported the mobile phone survey questions confusing and difficult to answer. One of the main misunderstandings was around the phrase “this morning 7 am going back to yesterday 7 am yesterday morning.” The participants suggested simplifying the phrase to “today,” “yesterday,” “last night,” “per day,” or “last week.” A few participants proposed changing the wording of the questions every so often so that they would not get bored of answering the same questions every time, as one participant said:

“I suggest if you change the questions… because if I know I'll be receiving the same SMS about the sex, obviously I'm going to be bored and sometimes I will ignore the phone because I know the questions. If you change the questions It will get much better.” (male, aged 19–30)

#### Timing of Mobile Survey Completion

Most participants wanted the mobile phone survey to be delivered weekly and sent on different days each week to avoid it from becoming predictable. Participants agreed that the assessment should be sent during the week because they were busier on the weekend and their phones were not always with them.

“At least Monday to Friday ‘cause for us on weekends we don't have our phones on us, obvious when you are places, the phone ends up lost.” (female, aged 19–30)

Three participants suggested that the survey be completed immediately after sex as it is easier to answer more accurately. One female participant mentioned that a daily survey be coitally dependent, that is completed soon after each sexual encounter:

“I prefer we answer when we have sex because if I'm not answering after I have sex I would be lying everyday…so I prefer when I have sex I will answer it” (female, aged 31–39)

Participants stated that five to 10 questions would be an appropriate length for the mobile phone survey and only one participant suggested that up to 15 would be acceptable.

#### Reminders

The majority of the participants agreed that a reminder would assist with completion of the mobile phone survey. Proposed modes of reminders included SMS and WhatsApp message and one participant mentioned a phone call. It was agreed that the reminders should be discrete to protect privacy. Suggestions of reminder content included a message that just mentions, ‘did you check your app [application] today?’ One participant mentioned that the message should be from a recognizable number and include a code word. There was no consensus on the best time of day to send the reminders and the number of the reminders that should be sent per day. The suggestions ranged from once a day at the same time to three times a day. However, it was stated that a reminder sent five times a day or after they had already completed the mobile phone survey would irritate the participants.

#### Privacy Settings

Privacy was a major concern for most of the participants. The biggest problem with using SMS or WhatsApp was the fear of lack of privacy.

“Yes on an app [application] because if it was a message like me I have four daughters and my first born likes my phone very much. You see if it comes with the messages this one will want to see and this one will also want to see but if it's an app [application] I would put in my password.” (female, aged 31–39)

“So you send me a message, then I reply to that message, meaning someone else can access [the message], then now I have the responsibility to delete the messages afterwards.” (male, aged 19–30)

Most agreed that a password protection would be adequate. Suggestions about the two ways in which this could be done are through a pin or login credentials.

“Creating credentials for logging in that would be good also, like nobody will be able to access it unless you and, the researchers.” (male, aged 19–30)

“I think, if you want to keep something secret for yourself others person can't go through your stuff before. Yes, you can have your own code because you gonna [going to] use your code to open and go through the app [application] so yes, okay, its good.” (male, aged 31–39)

Keeping the mobile phone survey confidential would facilitate its completion. When answering the assessment, it was important to the participants that it was confidential and they could not be recognized by name, especially considering the risk of it being hacked. The general consensus was that the participants did not mind answering intimate questions, especially if they had agreed to participate and the answers were kept private and confidential. One participant used the term anonymous but described confidentiality by stating:

“Plus, to add on that, one thing I actually saw is that it doesn't actually request your name. So, you might feel comfortable knowing that you [are] answering anonymously [confidentially].” (male, aged 19–30)

When talking about confidentiality, another participant agreed:

“I think it's the best thing ‘cause if you don't know my real name and you ask “did you have sex” I answer confidently “yes” … I will answer honestly.” (female, aged 31–39)

#### Incentives

Most of the participants agreed that airtime or data incentives between ZAR 5-10 (USD 0.29-0.58) would motivate them to complete the mobile phone survey, although one participant thought that the health benefit of the mobile phone survey would suffice.

“A little bit of motivation will do ‘cause you know, especially when you will be using the phone we send you 10 rand (USD 0.56) data … I know that every time I complete it I will have data for sure, there's no way I will never complete that survey.” (female, aged 19–30)

“ZAR 5 (USD0.29) airtime, I love airtime. If you say you're going to get R5 airtime I'm going to remember that [to complete the survey] every day.” (male, aged 19–30)

All participants agreed that completing the mobile phone survey should be free and any compensation should at least cover the data cost of participating. A concern raised about incentives was that they might encourage dishonesty to obtain the reward or may be misunderstood as a reward for having unprotected sex. Participants agreed that they would like to receive personalized feedback, through counseling or other formats, after completing the risk assessment survey to help them decrease their risk. They did not, however, mention on what platform they would prefer such feedback.

### Barriers to Long Term Use of a Mobile Phone Survey

Barriers were related to the frequency of completing mobile phone surveys, the amount of time it takes to complete the survey, and the cost of answering mobile phone surveys.

HIV prevention efficacy trials typically last more than 2 years and using the mobile phone survey during this time period was discussed with the participants. The participants were concerned that daily mobile phone surveys would be too frequent and might cause participants to opt out, answer insincerely, or miss days.

“You are supposed to answer according to the experience but human nature we've got those tendencies of saying I do every day and I won't answer for today I will answer tomorrow. So I think that's where the challenge will be. But of course it has to start with us, it's for our benefit as long as we remember that and be honest with it.” (female, aged 19–30)

Some participants were concerned that they would not have time to complete the mobile phone survey daily due to travel, socializing or work.

“The point you just made that the survey is for a month or 2 months. The problem I would have is time; you see? Because you would want to do the survey but you may have problems. As [another participant] has mentioned before life is very busy and as a person who loves to travel, even though you have the survey on your phone and there some things that we need to use our hands for. We always have our phones with us but the problem is actually completing the survey.” (male, aged 19–30)

It was important that the time taken to complete the mobile phone survey did not detract from daily activities and was short enough to accommodate their daily routines.

Another possible barrier to completing the survey was related to the cost. Depending on the format of the mobile phone survey, different costs may be incurred, for example if it is given using an SMS system one participant mentioned:

“…but again SMSs, a person never has airtime, SMSs need airtime and SMSs are expensive. Imagine answering 15 questions via SMS it's more than two pages when you check airtime gone.” (female, aged 31–39)

### Facilitators to Long Term Use of a Mobile Phone Survey

Facilitators included confidentiality when answering the mobile phone survey, the use of a study phone and the improved understanding of sexual risk that the mobile phone survey would enable. Most male participants agreed that it was preferable to use a study phone to complete the mobile phone survey in order to separate it from other parts of their life and to negate the need for them to use their own phone memory space. The female participants were divided, and some thought it would be enough to have a password protected application on their own phone. Participants raised a few concerns regarding the logistics of giving out study phones, including the scalability, lost or stolen study phones and the risks of phones being sold for profit.

“I think using the study phone would be convenient for everybody. … but on the very same breath, it will be harmful because possibilities are that I might lose the phone, get mugged. and then I lose the phone.” (male, aged 19–30)

“On my side it's fine with the study phone, because I have too much apps on my phone I don't even have a space anymore.” (male, aged 19–30)

Motivations that would facilitate the completion of the mobile phone surveys were to improve understanding of participant's own sexual behavior, to look after their health and to help the community. One participant (male, aged 31–39) mentioned that the mobile phone survey would be a “wake-up call” to them if they answered it just after “making a mistake.” Another felt that,

“It would help me in to understand what is happening in my life related to sex, like in a week maybe you had sex how many times and you used a condom how many times.” (female, aged 31–39)

## Discussion

Our findings provide user recommendations to optimize an existing mobile phone sexual risk assessment for use in HIV prevention clinical trials. In general, participants preferred a discrete and private password protected application with up to 10 simply worded sentences that could be offered in a range of South African languages. In the context of long-term HIV vaccine trials, participants suggested that mobile phone surveys should be completed weekly or soon after sexual activity with discrete message reminders to prompt completion. If the mobile phone surveys are given weekly, participants would prefer to complete them on weekdays, however, a study done in women with sexually transmitted diseases found that their sexual activity peaked on Fridays and Saturdays (36) and a literature review found that in some populations, HIV risk behavior had weekend patterns (37). This disconnect between what participants want and when they engage in sexual activity may lead to inaccurate information from the mobile phone survey due to recall bias. If the survey is sent early on in the week this could mitigate the potential issue. These practical recommendations are valuable considerations for those designing mobile health technologies targeted to sexual risk assessments.

Acceptance and use of mobile phone technology are improved when the applications are made with user preferences in mind (38, 39) and it is the hope that by optimizing the mobile phone surveys to accommodate the needs of the user, greater uptake will occur, thus improving the completion rates and data accuracy. For example, given the difficulty of participants understanding 24 h intervals such as “7 am today to 7 am yesterday,” making efforts to automate the calculations of time intervals may help with reporting accuracy.

Facilitators to completion of the mobile phone survey in the long term include confidentiality, where participants feel more comfortable answering intimate questions when they are unable to be identified. Confidentiality assists in the reduction of social desirability bias that is inherent in in-clinic assessments. The provision of study phones was also seen as a potential facilitator, especially among males. Interestingly, there was a similar finding amongst the study staff in the HVTN 915 study, where they recommended that study phones be given to participants in future studies (23). This strategy could be a costly addition to large HIV prevention clinical trials and more research into the feasibility of upscaling phone provision to participants needs to be done.

Incentives to complete the mobile phone surveys are necessary and airtime between ZAR 5-10 (USD 0.29-0.58) would suffice. Previous studies have included multiple incentives such as providing mobile phones and airtime to ensure adherence to the mobile phone surveys (25). HVTN 915 provided each participant with a mobile phone that they were able to keep after they had completed the study and gave them ZAR 5 (USD 0.5 at the time) airtime as an incentive to complete the mobile phone survey, to which they had a response rate of 82% to a daily mobile phone survey after 3 months (23). Some of the participants in this study were concerned that providing incentives could lead to dishonesty, but in HVTN 915 it was found that the mobile phone survey in combination with daily vaginal swabbing actually supported accurate reporting of protected and unprotected sex among young women (23, 25), even though multiple incentives were given.

Participants reported barriers to completing mobile phone surveys that need to be mitigated. Response fatigue to survey completion over years is one of them. HIV prevention trials are usually conducted over a long period of time and participants were concerned that completing the mobile phone survey frequently could become tiresome and lead to lower completion rates and possible attrition, which decreases the sample size and therefore power of HIV prevention trials and may introduce bias (40). There were similar findings in studies conducted by Dietrich et al. (23) and Curran et al. (24) where response rates to mobile phone surveys decreased in the later stages of the trials. Incentives and reminders are two methods that have been used to reduce this problem. Another creative solution that emerged was to introduce an element of variation into both the content and wording of the mobile phone surveys and reminders to keep the process interesting, thus potentially reducing the response fatigue. However, asking questions differently will prevent investigators from monitoring responses over time, as differently worded questions may lead to different understanding and potentially different answers (41, 42). In addition, incentives could be increased over the study period to support survey completion. The monetary and time costs of completing the mobile phone survey are important barriers that need to be addressed. A pilot study done by Mngadi et al. investigating feasibility and acceptability of using USSD to assess reactogenicity symptoms in a HIV vaccine trial ensured that all costs of the tool were directly charged to the research site and that participants would be able to access the tool even if they did not have airtime (43). This method of removing any monetary costs to the participant could be an effective way of reducing this barrier. If an SMS system is used, an effective way to minimize the monetary cost is to ensure that all the survey answers are transmitted in a single SMS as opposed to one SMS per answer. In order to mitigate the time cost, mobile phone surveys should be kept short and ensure that the format is compatible with completion in any setting.

These data have provided key recommendations for adapting our existing mobile phone survey as well as facilitators and barriers to be considered when we implement it. More research needs to be done with a larger sample size once the mobile phone survey is optimized using these recommendations.

### Limitations

The findings in this paper are from a small sample which can not necessarily be applied to all people of similar demographics. In addition, the study was conducted outside of an existing clinical trial and, although the gender and ages of the participants are similar to those in the trial, no data were collected on sexual risk and health for the participants of this study and they may differ in some ways to those participating in HIV prevention clinical trials, such as HIV risk.

There was a disconnect between what the mobile phone survey was designed to do (that is, collect data on participant's sexual activity) and what participants thought it should do. Some participants reported a desire to receive feedback on their sexual risk but this was not the intended aim of the mobile phone survey and its design. There is an HIV risk prediction tool that has successfully been developed and validated in men who have sex with men in the United States of America (44) and work is being done on the development of an HIV risk calculator for young South Africans (45). Future studies could incorporate these tools whilst taking trial design limitations into account.

A strength of the study was that we collected quantitative and qualitative data. One of the limitations with this, however, was that the quantitative data were only used to collect information on demographics and access to mobile phones and internet, and not on the user recommendations. Another strength of the study was that recommendations were based on an already developed mobile phone application and not a hypothetical application and this allowed participants to interact with the mobile phone survey before discussing the recommendations, facilitators and barriers.

### Conclusions and Implications

Our findings provide a user-centered approach to application design and development of conducting behavioral risk assessments in HIV prevention research. User recommendations have the potential to be used not only to improve our mobile phone sexual risk assessment but for other mHealth strategies used in HIV prevention programmes and research in South Africa. In terms of practical implications, findings from this study will be used to optimize our existing application-based mobile phone sexual risk assessments. This method of obtaining remote sexual risk data from trial participants is of particular relevance in the present context of COVID-19 physical distancing. Further research is needed to determine how men in South Africa would adhere to completing mobile phone sexual risk assessments. Further, the literacy levels and technology use of older groups in South Africa may be different to the young women who participated in HVTN 915. Therefore, further investigation could provide data on the acceptability and feasibility of conducting mobile phone sexual risk assessments in older South Africans.

## Data Availability Statement

The datasets presented in this article are not readily available because transcripts may contain identifiable data. Requests to access the datasets should be directed to Janan J. Dietrich, dietrichj@phru.co.za.

## Ethics Statement

The studies involving human participants were reviewed and approved by the University of the Witwatersrand, Human Research Ethics Committee. The participants provided their written informed consent to participate in this study. Written informed consent was not obtained from the individual(s) for the publication of any potentially identifiable images or data included in this article.

## Author Contributions

JJD conceived and designed the study, participated in study procedures, data analysis, interpretation, and manuscript writing. GLB conducted data analysis and interpretation, drafted the manuscript, and contributed to revising the manuscript critically. MM conducted data analysis and interpretation and contributed to manuscript writing. AK contributed to the development of study materials and manuscript writing. SH developed the protocol for IRB approval and contributed to manuscript writing. LMM was responsible for data collection and contributed to manuscript writing. MPL contributed to study design, data interpretation, and manuscript writing. EL contributed to data interpretation and manuscript writing. MPA contributed to data analysis and interpretation, as well as to manuscript writing. KJH supervised JJD with regard to digital health, contributed to study materials, data analysis, interpretation, and contributed to manuscript writing. All authors discussed the results and contributed to the final manuscript.

## Conflict of Interest

The authors declare that the research was conducted in the absence of any commercial or financial relationships that could be construed as a potential conflict of interest.

## Footnotes

^1^Emoji's are small icons or images used in electronic communication that are used to express emotions or ideas (35).
