# Supplementary material for: “You Are on the Right Track With the App:” Qualitative Analysis of Mobile Phone Use and User Feedback Regarding Mobile Phone Sexual Risk Assessments for HIV Prevention Research
Source: Front Digit Health. 2021 Mar 22;3:576514. doi: 10.3389/fdgth.2021.576514 (PMC8521804; doi:10.3389/fdgth.2021.576514)
Supplement: Supplementary file 1 [file Data_Sheet_1.PDF]

Interview Guide for Focus Group Discussions

Title: User friendly technologies for HIV vaccines clinical trials: Adapting a mobile phone risk assessment tool for adult men and women in Soweto, South Africa

Investigator: Dr. Janan Dietrich

Note: This is a semi-structured interview guide which may change based on further literature searches and focus group discussions feedback.

Introduction

Hi everyone. Welcome to the focus group. Thank you for taking the time to be here.

[Introduce team members]

Before we start, we want to have a few group rules:

- We want to make this a safe space for everyone. Please only use the name you choose when you filled out the form earlier.
- When you talk, please state the name you choose for this group discussion, so we know later on who was speaking. I'll remind you about that as we begin.
- People have different opinions about things. Please share your opinion and respect the opinions of others.
- What is said in this room stays here. Please respect the right of everyone to privacy.
- We only have a short time together, so if we're spending too much time on a topic, I'll ask to move to the next topic. I'm not intending to be disrespectful, but I want to make sure we're getting to all of the topics.
- We will have a discussion for about 1h30 with breaks in between.
- Remember to have fun. This is meant to be informal and we're just asking for your opinion. We'd love to hear from you, but will not force anyone to talk.
- It's ok to respond to each other's comments. If someone says something that you have some thoughts on, please say something and remember to mention your name before you speak.
- Finally, we want to make sure that it's ok to audio record this focus group. This is only because we can't take notes fast enough to capture everything you're sharing with us. So, this will allow us to go back and review what we talked about. When you talk, please remember to use only the name you have chosen for this focus group. Please don't use your real name. Is it ok to begin the recording?

Any questions?

Overview of Focus Group

Here's what I'd like to cover today:

1. First I'll ask some questions about your technology use in your daily life, such as using a mobile phone and the internet.

2. Then we will talk about your own impressions and experience with the mobile survey we will show you today.

3. Last we would like to talk with you about your thoughts on creating a data collection survey to collect data on intimate relationships.

Part 1: General technology use questions about mobile phone use and internet
Let's start with talking about mobile phones and how we can use them to collect information.

1. What kind of mobile phone do you have?
   Probe: [give phone brand and make (e.g. Samsung Galaxy Star, BlackBerry etc.) and type of phone (Smartphone (has internet access) vs. Basic phone (can only make calls and write SMS)]

2. If you do not have a phone but need to use one, what do you do?
   Probes:
   - *Do you borrow someone else's phone? What are some of the advantages and disadvantages of using someone else's phone?*

3. What do you primarily use your mobile phone for?
   Probes:
   Making calls, SMS, accessing the internet, using apps, Music, WhatsApp, Facebook etc.

4. Do you use your phone to use the internet or do you access the internet in some other way?
   Probes:
   - *If they go onto the internet, what type of heath information do you look for online*

   - *[For smartphone users] What Apps do you use on your smartphone?*

Part 2: Experience with the mobile phone survey
Now we are going to talk about your experiences with the mobile phone survey that you have completed earlier.

1. Tell me about your experiences with the **survey application** and the **smart phone** that we provided to you. Was it easy to use? Why/why not?
   Prompts: the way it was set-up, user-friendliness

2. Now we are talking about the survey layout and questions. I will show you the questions again that we included in the survey that ask about intimate relationships and I would like to ask you to give me feedback on the wording, content and layout.

- What do you think about the questions? Would you change anything? (*Prompts: amount of questions, phrasing)*
- What should the survey look like *(Prompts: design appearance, layout, colours, pictures, set up)*
- What languages do you think we should use for the messages? (English or home language),
- Would you use formal/informal content *(Prompts: e.g. modern language, such as LOL, smileys?)*

3. If we asked you to complete the survey over a long period of time – let's say 2 months – what problems do you think you would have completing the survey for that period? (e.g. 8 weeks?
Prompts: privacy issues, partner disclosure to study, housing conditions, electricity, network reception, boring, life gets in the way, loose interest

4. Should we use the same survey app we have showed you earlier that works via the internet? Why/why not?

5. If you were going to answer this survey every day for a month or more, what are your concerns about having access to the survey the entire time? What are your concerns about privacy (e.g. other people close to you seeing the survey questions during the study participation)

6. How helpful are text message (SMS) reminders to remind you to complete the survey? How many reminders would you prefer to receive in a day? What is the best time for you to receive the SMS reminders? What would you like the reminders to say?

Part 3: General feedback on creating a data collection programme for data collection on intimate relationships

For the last set of questions, we would like to get your opinion about creating a data collection programme to collect data on intimate relationships.

7. How do you feel about receiving short surveys over your mobile phone in general? Would you prefer to receive surveys using text messages or through a mobile app? Why?

8. What are other ways can we collect data on intimate relationships via a mobile phone? What do you think would work best? *(Probe: SMS, games, social networking sites etc.)*

9. Tell us about different types of incentives you would like to receive for completing the survey on the mobile phone. What would you like to receive? How frequent? *Prompts: Airtime, data, cash, prizes, value; frequency: daily, weekly, monthly etc.*

In the future, we are hoping to reach men and women to get information on their intimate relationships via a mobile phone. What ideas do you have for reaching men and women in your community about this

10.

11. mobile phone programme?

Probes:
How can we include young women and men who do not have their own mobile phones? How can we include women and men who do not have access to a mobile phone?

12. We realize that many phones that people have in Soweto are not internet enabled, in some studies we would be able to give people a phone. How would you feel having two phones with you (your own and study phone)? Would it be easy or difficult to look after two phones and to carry them with you all the time? What are the pros and cons?
