# Supplementary material for: “You Are on the Right Track With the App:” Qualitative Analysis of Mobile Phone Use and User Feedback Regarding Mobile Phone Sexual Risk Assessments for HIV Prevention Research
Source: Front Digit Health. 2021 Mar 22;3:576514. doi: 10.3389/fdgth.2021.576514 (PMC8521804; doi:10.3389/fdgth.2021.576514)
Supplement: Supplementary file 2 [file Data_Sheet_2.PDF]

Study Number ☐ ☐

DEMOGRAPHIC QUESTIONNAIRE

Personal Information

1. **Age** ______ yrs

2. **Gender**
   ☐ Male
   ☐ Female

3. **Where do you live in Soweto?** ___________________________________

4. **main language is spoken in your home? (please choose only one)**

Afrikaans	☐	IsiZulu	☐	SiSwati	☐
English	☐	Northern Sotho	☐	Tshivenda	☐
IsiNdebele	☐	Sesotho	☐	Xitsonga	☐
IsiXhosa	☐	Setswana	☐		

Other (please specify) __________

5. **What is the main material that the walls of your house are built of? (please choose only one)**

☐ Brick house owned by family
☐ Brick house that family is renting
☐ Flat owned by family
☐ Flat that family is renting
☐ RDP house
☐ Hostel (Brick)
☐ Shack - Informal settlement
☐ Shack – Backyard
☐ Other, Specify _____________________

Study Number ☐ ☐

6. **What is the highest level of formal education you have completed?** (please choose only one*)*

- ☐ No formal education
- ☐ Incomplete primary school (up to grade 7)
- ☐ Complete primary school (completed grade 7)
- ☐ Incomplete high school (up to grade 12)
- ☐ Complete high school (completed grade 12)
- ☐ Incomplete post-high school training (Trade or technical training, college, or university)
- ☐ Complete post-high school training (Trade or technical training, college, or university)
- ☐ Other (*please specify*): ______________________

Mobile Phone Usage

7. **Do you own a personal mobile phone?**

☐ Yes
☐ No

8. **If YES to question 7, do you share your personal phone with someone?**

☐ Yes
☐ No

9. **If NO to Question 7, do you use some else's mobile phone?**

☐ Yes
☐ No

10. **Iif YES to 8 or 9, with who do you share your phone with? (choose all that apply)**

☐ Parents
☐ Partner
☐ Sibling(s)
☐ Friend(s)
☐ Grandparents
☐ Cousin
☐ Other (please specify): ______________________

11. If YES to 7 or 9, what type of phone do you have/ share?

- ☐ Samsung
- ☐ BlackBerry
- ☐ Nokia
- ☐ Sony
- ☐ LG
- ☐ HCT
- ☐ Huawei
- ☐ Motorola
- ☐ Other (please specify): ________________________

12. If YES to 7 or 9, please specify the make of the phone (e.g. Huawei P10):

____

- ☐ Don’t know

13. Can you access the internet on the phone?

- ☐ Yes
- ☐ No

14. Can you download Apps with the phone?

- ☐ Yes
- ☐ No

15. **What do you use the mobile phone for?** *(*please read list and choose all that apply*)*

- ☐ Send and receive SMS
- ☐ For emergencies
- ☐ Making phone calls
- ☐ Receiving phone calls
- ☐ Listening to music/radio
- ☐ Play games
- ☐ Access the internet
- ☐ Facebook
- ☐ Twitter
- ☐ Instagram
- ☐ WhatsApp
- ☐ We Chat
- ☐ YouTube
- ☐ Cellphone banking

☐E-mail
☐Google Playstore
☐App Store
☐Other (*please specify*): ______________________

16. How much time in a day do you spend actively using a mobile phone? *This includes using it for listening to music/radio, SMS, making phone calls, playing games and accessing the internet.*

☐0-1 hours
☐2-4 hours
☐5-7 hours
☐More than 8 hours
☐Don't know

17. How do you get airtime?

☐Prepaid
☐Contract
☐I don't get airtime
☐Don't know

18. Who pays for the airtime?

☐Parent
☐Friend
☐Partner
☐My own money
☐Sibling
☐Other (please specify): ______________________

19. How do you get data bundles?

☐Prepaid
☐Contract
☐I don't get data
☐Don't know

Study Number ☐ ☐

20. Who pays for the data?

☐Parent

☐Friend
☐Partner
☐My own money
☐Sibling
☐Other (please specify): ______________________

21. In the past year, was your phone service ever disconnected because you could not pay the bill, afford airtime or because your phone was lost or stolen?

☐Yes
☐No

<u>Internet Access and Usage</u>

22. In the last 6 months, have you had access to the internet?

☐Yes
☐No

23. **How do you mainly access the internet?** (please choose only one)

☐ Mobile Phone
☐ PC
☐Laptop
☐ Tablet
☐Other (please specify): ___________________

Study Number ☐ ☐

24. What are the reasons you are using the internet? (please read list and choose all that apply)

☐ To make new friends
☐ To chat with friends
☐ To chat with family
☐ Download music and videos
☐ Research for school/work projects
☐ Look for work opportunities
☐ Dating sites
☐ Meet partners for sex
☐ To get directions to places (GPS) and look at maps
☐ To find information about health
☐ To find information about drugs or alcohol
☐ To find information about HIV/AIDS
☐ To find information about intimate relationships
☐ To find information about depression, anxiety or suicide
☐ To find out about parties/ DJ events
☐ Internet banking
☐ To use social media sites (e.g., Facebook, Twitter, Instagram etc.)
☐ E-mail
☐ Selling and buying sites (e.g. Olx, GumTree or Bid or Buy)
☐ Other (please specify): ______________________

25. Do you have an active Facebook profile?

☐ Yes
☐ No
